# Nucleosome stability safeguards cell identity, stress resilience and healthy aging

**DOI:** 10.21203/rs.3.rs-7913970/v1

**Published:** 2025-11-18

**Authors:** Peter Adams, Hiroshi Tanaka, Brenna McCauley, Clara Guida, Xue Lei, Sha Li, Tatiana Moreno, K’leigh Guillotte, Zong Chua, Adrianna Abele, Aashna Lamba, Rouven Arnold, Adarsh Rajesh, Marcos Teneche, Laurence Haddadin, Anagha Deshpande, Aniruddha Deshpande, Alexandre Colas, Caroline Kumsta, Michael Petrascheck, Rolf Bodmer, Weiwei Dang

**Affiliations:** Sanford Burnham Prebys Medical Discovery Institute; Sanford Burnham Prebys Medical Discovery Institute; Baylor College of Medicine; Sanford Burnham Prebys Medical Discovery Institute; Sanford Burnham Prebys MDI; Sanford Burnham Prebys Medical Discovery Institute; Sanford Burnham Prebys Medical Discovery Institute; Sanford Burnham Prebys Medical Discovery Institute; Sanford Burnham Prebys Medical Discovery Institute; Sanford Burnham Prebys Medical Discovery Institute; Sanford Burnham Prebys Medical Discovery Institute; Sanford Burnham Prebys Medical Discovery Institute; Sanford Burnham Prebys Medical Discovery Institute; Sanford Burnham Prebys Medical Discovery Institute; Sanford Burnham Prebys Medical Discovery Institute; Sanford Burnham Prebys Medical Discovery Institute; Sanford Burnham Prebys Medical Discovery Institute; Sanford Burnham Prebys Medical Discovery Institute; Sanford Burnham Prebys Medical Discovery Institute; The Scripps Research Institute; Sanford Burnham Prebys Medical Discovery Institute; Baylor College of Medicine

## Abstract

The Information Theory of Aging (ITOA) proposes that aging results from the progressive loss of epigenetic information. As the repeating units of the epigenome, nucleosomes are considered pivotal for its stability. Accordingly, the ITOA predicts that destabilization of nucleosomes will accelerate aging. However, this causal link has not been directly tested. Here, we addressed this through histone mutants that weaken histone–histone interactions. Without broadly perturbing steady-state chromatin accessibility, DNA damage, cell proliferation or viability, nucleosome instability compromised cell identity maintenance, altered lineage specification and activated intrinsic inflammatory and stress pathways in a manner reminiscent of aging in mouse tissues and human cells. Consistently, nucleosome instability accelerated age-associated transcriptional alterations and functional decline in Caenorhabditis elegans and Drosophila melanogaster, and reduced cellular resilience to exogenous perturbations—including environmental, epigenetic and mitotic stress—in human cells and Saccharomyces cerevisiae. These cross-species findings establish nucleosome stability as a fundamental requirement for preserving cell identity and stress resilience, thereby safeguarding organismal longevity.

## Introduction

The Information Theory of Aging (ITOA) proposes that aging results from the progressive loss of epigenetic information that defines and maintains cell identity and function, originally established during development^1^. This concept is supported by multiple lines of evidence: (1) epigenetic changes progressively accumulate over time, underpinning epigenetic clocks^2–4^; (2) perturbations in chromatin modifiers can causally accelerate or delay aging phenotypes^5–7^; and (3) reversal of age-associated epigenetic changes can restore cellular or tissue function at least in certain contexts^8–10^. In this framework, the epigenome acts as an information system whose stability ensures accurate execution of genomic processes—such as transcription, DNA replication and repair and mitosis—thereby preserving cell identity and stress resilience throughout life.

Central to the ITOA are nucleosomes—the minimal repeating units of chromatin—whose dynamic assembly and disassembly underpin epigenomic stability^11–16^. Each nucleosome consists of ~147 bp of DNA wrapped around a histone octamer (two copies each of H2A, H2B, H3 and H4), whose intrinsic stability depends on defined histone–histone and histone–DNA interactions^17^. Although the ITOA suggests that destabilization of nucleosomes may jeopardize the long-term storage of epigenetic information and accelerate the rate of information loss—and hence aging—this hypothesis has not yet been directly tested. Intriguingly, cancer-associated somatic mutations in histones—such as H2B D68 and E76—frequently target residues within the histone–histone interface that are critical for nucleosome stability^18–22^, suggesting that nucleosome destabilization may intrinsically threaten cell identity and promote phenotypic drift without causing catastrophic damage. To test the ITOA by manipulating nucleosome stability, we introduced defined histone mutants that destabilize nucleosomes into diverse biological systems, including cultured cells, tissue models and whole organisms. This cross-model approach allowed us to examine whether nucleosome destabilization is sufficient to perturb cell identity, impair stress resilience and undermine healthy aging.

## Results

### Characterization of nucleosome-destabilizing histone mutants

**Figure 1:**
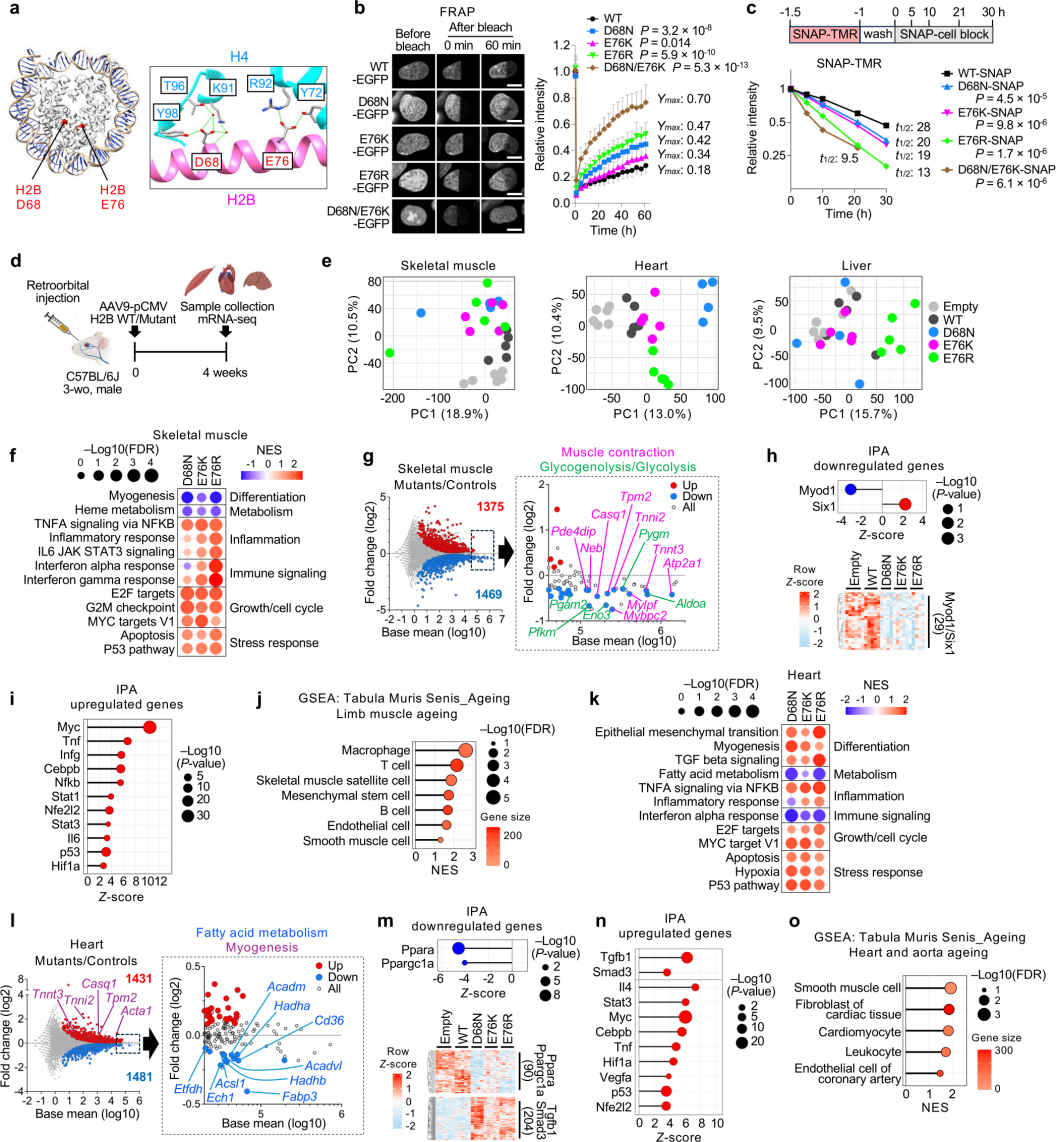
Nucleosome instability destabilizes cell identity and activates stress, inflammatory and immune responses reminiscent of aging in mouse tissues. **a,** Nucleosome structure (PDB: 3AFA), highlighting H2B residues D68 and E76, which were mutated in this study. H2B is shown in magenta, H4 in cyan. **b,** FRAP analysis of IMR-90 SV40-T cells expressing EGFP-tagged H2B WT or mutants. Scale bar, 10 mm. Relative intensity was calculated by comparing the bleached region to the unbleached region. Data represent mean ± s.d. from >15 nuclei. The plateau value (*Ymax*) was calculated from the fitted recovery curve. *P*-values were calculated using unpaired, two-tailed Student’s *t*-tests comparing each mutant to WT at the final time point. **c,** Pulse-chase labeling of SNAP-tagged H2B WT or mutants using SNAP-TMR. Data represent mean ± s.d. of total field fluorescence from >2,600 cells across three independent wells per condition. Half-life (*t*_*1/2*_) was calculated from the fitted decay curve. *P*-values were calculated using unpaired, two-tailed Student’s *t*-tests comparing each mutant to WT at the final time point. **d,** Schematic of the experimental design. C57BL/6J male mice were retro-orbitally injected with AAV9-pCMV vectors expressing H2B WT, mutants (D68N, E76K, E76R) or an empty vector. Tissues were collected 4 weeks post-injection. **e,** PCA of bulk mRNA-seq from skeletal muscle, heart and liver expressing H2B WT, mutants or empty vector. Each dot represents an individual mouse (n = 6, Empty; 5, WT; 4, D68N; 5, E76K; 5, E76R). **f,** GSEA of hallmark pathways in transduced skeletal muscle comparing each mutant to controls. See Extended Data Fig. 3c for full pathway list. **g,** MA plot of skeletal muscle comparing mutants to controls. Genes involved in myogenesis-related pathways, including muscle contraction and glycogenolysis/glycolysis, are highlighted. **h,i,** IPA upstream regulator analysis showing suppression of Myod1- or Six1-associated genes and activation of inflammatory and stress-response genes in mutant-expressing skeletal muscle compared to controls. **j,** GSEA of Tabula Muris Senis aging signatures in transduced skeletal muscle comparing mutants to controls. **k,** GSEA of hallmark pathways in transduced heart tissue comparing each mutant to controls. See Extended Data Fig. 3h for full pathway list. **l,** MA plot of heart tissue comparing mutants to controls. Genes involved in fatty acid metabolism and myogenesis are highlighted. **m,n,** IPA upstream regulator analysis showing suppression of Ppara- or Ppargc1a-associated genes and activation of Tgfb1- or Smad3-associated, inflammatory and stress-response genes across mutant-expressing heart compared to controls. **o,** GSEA of Tabula Muris Senis aging signatures in transduced heart tissue comparing all mutants to controls.

As tools to probe the impact of nucleosome instability on cell phenotypic stability and aging, we employed histone H2B mutants that disrupt histone–histone interactions within the nucleosome. The D68 residue of H2B forms hydrogen bonds with K91, T96 and Y98 of one H4 molecule, while E76 interacts with Y72 and R92 of the other H4 (Fig. 1a). Hence, mutations at these residues within H2B disrupt H2B–H4 interactions and confer nucleosome instability^18,20,23^. We transduced immortalized IMR-90 human fetal lung fibroblasts (IMR-90 SV40-T) with lentiviral vectors expressing either wild-type (WT) or mutant H2B (D68N, E76K, E76R or double mutant D68N/E76K) (**Extended Data Fig. 1a–c**). Although the E76K and E76R substitutions both replace acidic E with basic K or R, the latter is more basic and bulkier and thus predicted to be more disruptive of nucleosome structure. Expression levels of H2B D68N and E76K were comparable to those of WT and similar to endogenous H2B, whereas E76R and D68N/E76K showed slightly lower expression, possibly linked to their heightened nucleosome instability. Expression of these mutants did not overtly affect cell proliferation or steady-state abundance of gH2AX, a marker of DNA damage (**Extended Data Fig. 1d,e**). EGFP-tagged WT and mutant H2B exclusively localized to the nucleus and were incorporated into mitotic chromosomes (**Extended Data Fig. 1f,g**), confirming their association with chromatin. However, although sequential salt fractionation showed that both WT and mutant H2B localized in the chromatin fraction, the mutants were extracted at a lower salt concentration (0.9 M NaCl) compared to endogenous H2B and ectopic H2B WT (1.2 M NaCl), indicating weaker chromatin association (**Extended Data Fig. 1h**). Consistently, fluorescence recovery after photobleaching (FRAP) demonstrated increased mobility of mutant proteins in the nucleus compared to WT (Fig. 1b). As predicted, E76R and D68N/E76K conferred higher mobility compared to E76K. Using EGFP-H2A, we also observed increased H2A mobility in the presence of H2B mutants, consistent with the destabilization of the H2A–H2B dimer in chromatin (**Extended Data Fig. 1i**). Complementing these findings, pulse-chase labeling of SNAP–tagged H2B showed faster turnover of mutant proteins compared to WT (Fig. 1c and **Extended Data Fig. 1j,k**). These results indicate that although both WT and mutant H2B are similarly incorporated into chromatin, the mutants induce nucleosome instability in cells.

### Nucleosome instability destabilizes cell identity and triggers aging-like transcriptional programs in mouse tissues

To assess the impact of nucleosome instability on tissue homeostasis, we retro-orbitally injected AAV9 vectors encoding WT or mutant H2B (D68N, E76K or E76R) under the CMV promoter into 3-week-old C57BL/6J mice (Fig. 1d). Four weeks later, body weight and gross appearance were unaffected, and expression of WT and mutant H2B was detected in skeletal muscle, heart and liver at the expression levels <50% of endogenous H2B (**Extended Data Fig. 2a–d**; see total H2B Western blot in 2b). Principal component analysis (PCA) and differentially expressed gene analysis on mRNA-seq revealed tissue-specific effects of H2B mutants: liver showed only modest changes, whereas skeletal muscle and heart each exhibited clear separation between mutant (D68N, E76K, E76R) and control (Empty, WT) samples, with each displaying >1,300 differentially expressed genes, both up- and downregulated (Fig. 1e and **Extended Data Fig. 3a,b**). Gene set enrichment analysis (GSEA) revealed repression of myogenesis and heme metabolism genes in mutant-expressing muscles, alongside activation of inflammatory, immune, growth and stress-response genes (Fig. 1f and **Extended Data Fig. 3c**). Indeed, genes associated with muscle contraction (e.g., *Atp2a1*, *Tnnt3*, *Tnni2*, *Tpm2* and *Casq1*) and glycolysis (e.g., *Aldoa*, *Eno3*, *Pgam2* and *Pfkm*) were significantly downregulated in mutant-expressing muscles (Fig. 1g). Ingenuity Pathway Analysis (IPA) inferred suppression of Myod1- and Six1-regulated myogenic programs (Fig. 1h). Simultaneously, it indicated activation of inflammatory, immune and stress pathways driven by Tnf, Nfkb, Cebpb, Il6, Stat3, Ifng, Stat1, Hif1a, Nfe2l2, Myc and p53 (Fig. 1i). In line with immune activation, chemokines *Ccl22*, *Ccl12* and *Cxcl10*, known to recruit macrophages and T cells and accumulate during muscle aging and regeneration^24,25^, were upregulated, accompanied by enrichment of proinflammatory M1 macrophage transcriptional traits (**Extended Data Fig. 3d,e**). Upregulation of p53 target genes (e.g., *Bbc3/Puma*, *Bax* and *Cdkn1a/p21*), muscle regeneration-associated genes (e.g., *Tceal7*, *H19* and *Pim1*)^26–28^ and Myc target ribosomal protein genes (RPLs and RPSs) suggests maladaptive regenerative responses reminiscent of aged muscle^29^ (**Extended Data Fig. 3f,g**). Cell type signature profiles using the mouse aging cell atlas, *Tabula Muris Senis*^30^, revealed that mutant-expressing muscle recapitulated aging-like transcriptional signatures across multiple cell types, including macrophages, T cells, muscle satellite cells, mesenchymal stem cells and B cells (Fig. 1j).

In the heart, mutants repressed fatty acid metabolism genes, while activating epithelial-mesenchymal transition (EMT), myogenesis, TGFb signaling, inflammation, growth and stress-response genes (Fig. 1k and **Extended Data Fig. 3h**). Strikingly, genes involved in fatty acid metabolism (e.g., *Cd36*, *Fabp3*, *Acsl1*, *Acadvl* and *Hadha*) were globally suppressed in mutant-expressing hearts (Fig. 1l). IPA predicted suppression of Ppara and Ppargc1a, master regulators of fatty acid metabolism^31^, while activating the TGFb1–Smad3 axis, a central mediator of EMT and myogenic processes^32^, as well as inflammatory, immune and stress pathways driven by Tnf, Cebpb, Il4, Stat3, Hif1a, Nfe2l2, Vegfa, Myc and p53 (Fig. 1m,n). Unlike skeletal muscle, mutant hearts upregulated the anti-inflammatory cytokine *Ccl17*, which plays a critical role in heart failure and cardiovascular aging^33^ (**Extended Data Fig. 3i**), suggesting tissue-specific immune adaptation. Activation of proliferative and growth programs, including ribosomal protein genes, suggested regenerative activity or hypertrophic growth responses under stress (**Extended Data Fig. 3j**). Cell type signature profiles showed patterns reminiscent of aging across smooth muscle cells, fibroblasts, cardiomyocytes, leucocytes and endothelial cells (Fig. 1o). Collectively, these results demonstrate that nucleosome instability impairs maintenance of tissue-specific cell identity programs, while activating persistent inflammatory, immune and p53-associated stress responses, thereby recapitulating aging-like transcriptional signatures across multiple tissues.

### Nucleosome instability compromises lineage specification in mouse HSPCs

**Figure 2:**
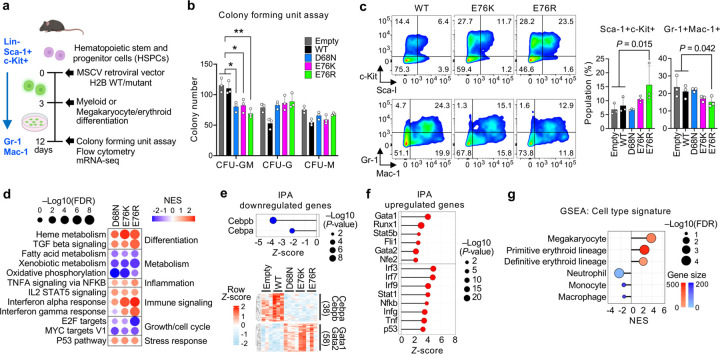
Nucleosome instability compromises lineage specification and biases transcriptional programs toward aging-like signatures in mouse HSPCs. **a,** Schematic of the experimental design. MSCV-based retroviral vectors expressing H2B WT or mutants were introduced into isolated HSPCs (Lin^−^Sca-1^+^c-Kit^+^), and myeloid or megakaryocyte/erythroid differentiation was assessed. **b,** Quantification of CFU colony types. Data represent mean ± s.d. for each condition. *P*-values were calculated using one-way ANOVA followed by Dunnett’s post-hoc test to compare each mutant with controls. *, *P* < 0.01; **, *P* < 0.001. **c,** Flow cytometry of Sca-1^+^c-Kit^+^and Gr-1^+^Mac-1^+^ cells after 9 days of differentiation. *P*-values were calculated using one-way ANOVA followed by Dunnett’s post-hoc test to compare each mutant with controls. **d,** GSEA of hallmark pathways in transduced HSPCs comparing each mutant to controls. See Extended Data Fig. 4c for full pathway list. **e,f,** IPA upstream regulator analysis showing suppression of Cebpa- or Cebpb-associated genes (myeloid regulators) and activation of Gata1- or Gata2-associated genes (megakaryocyte/erythroid regulators), as well as inflammatory and stress-response genes in mutant-expressing HSPCs after 9 days of differentiation. **g,** GSEA of cell type signatures for megakaryocyte, erythroid and myeloid (neutrophil, monocyte and macrophage) features in mutant-expressing cells compared to controls.

Given that nucleosome instability perturbs cell identity programs and induces aging-like features in mouse tissues, we next investigated its direct impact on lineage specification in mouse hematopoietic stem and progenitor cells (HSPCs). Hematopoiesis is a dynamic process in which HSPCs commit to specific blood cell lineages in response to differentiation cues^34,35^, and aging reduces this capacity while skewing output toward myeloid and megakaryocyte/erythroid lineages^36–39^. Freshly isolated mouse HSPCs (Lin^−^Sca-1^+^c-Kit^+^) were transduced with MSCV retroviral vectors expressing H2B WT or mutants and cultured in semi-solid methylcellulose medium to induce myeloid and megakaryocyte/erythroid differentiation (Fig. 2a and **Extended Data Fig. 4a**). After 8 days, three colony types were detected: colony-forming unit granulocyte (CFU-G), macrophage (CFU-M) and granulocyte/macrophage bipotent progenitors (CFU-GM) (**Extended Data Fig. 4b**). Notably, H2B mutants markedly reduced CFU-GM formation (Fig. 2b). Consistently, flow cytometry showed a trend toward retention of Sca-1^+^c-Kit^+^ undifferentiated cells and a reduction in Gr-1^+^ and Mac-1^+^ myeloid lineage cell populations, with a significant effect observed in E76R (Fig. 2c), suggesting impaired HSPC differentiation.

Transcriptomic profiling after differentiation revealed downregulation of metabolic pathways, including fatty acid metabolism, xenobiotic metabolism and oxidative phosphorylation, as well as genes associated with growth and cell cycle (Fig. 2d and **Extended Data Fig. 4c**). These changes are consistent with the observed retention of undifferentiated stem cells, as both mitochondrial energy metabolism and proliferation are critical for HSPC lineage specification^40^. Concurrently, heme metabolism, TGFb signaling, inflammation, interferon and p53 pathway genes were upregulated (Fig. 2d). IPA indicated suppression of Cebpa and Cebpb—key drivers of myeloid specification^41,42^—and activation of Gata1, Gata2, Runx1, Nfe2, Fli1 and Stat5b, which promote heme metabolism and megakaryocyte/erythroid differentiation^43–45^ (Fig. 2e,f and **Extended Data Fig. 4d**). Consistently, cell type signature profiles indicated loss of neutrophil, monocyte and macrophage signatures, whereas megakaryocyte and erythroid signatures were enriched (Fig. 2g). This skewed output was accompanied by activation of inflammatory and interferon signatures (Tnf, Nfkb, Irf3/7/9, Ifng, Stat1) and p53 pathways—known to impair HSPC differentiation and promote lineage bias^46–48^ (Fig. 2f). Together, these data demonstrate that nucleosome instability compromises the intrinsic differentiation potential of HSPCs by impairing lineage-determining transcriptional programs and biasing transcriptional output toward megakaryocyte/erythroid lineages, partially recapitulating signatures of hematopoietic aging.

### Nucleosome instability drives aging-like identity remodeling and stress signaling in human fibroblasts

**Figure 3:**
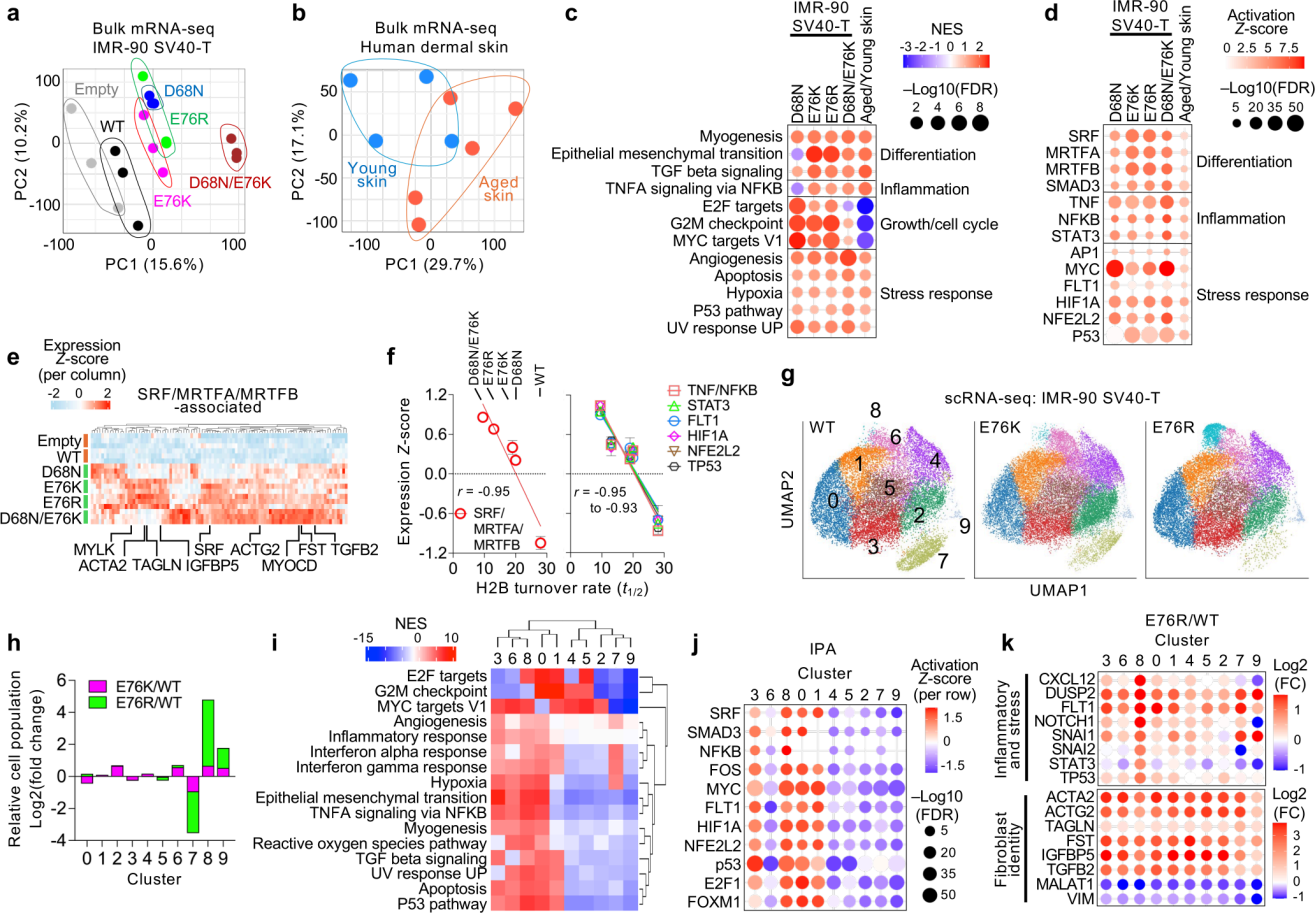
Nucleosome instability drives aging-like identity and stress remodeling toward a myofibroblast-like state in human fibroblasts at single-cell resolution. **a,** PCA of bulk mRNA-seq from IMR-90 SV40-T cells expressing H2B WT, mutants or empty vector. Each dot represents an individual replicate (n = 3 per condition). **b,** PCA of bulk mRNA-seq from dermal skin biopsies comparing young (ages 23–33, n = 4) and aged (ages 64–72, n = 5) individuals. **c,** GSEA of hallmark pathways in mutant-expressing IMR-90 SV40-T cells compared to Empty and WT cells and in aged primary skin fibroblasts compared to young fibroblasts. See Extended Data Fig. 5a for full pathways. **d,** IPA upstream regulator analysis showing activation of differentiation, inflammation and stress-response pathways in mutant-expressing IMR-90 SV40-T cells compared to Empty and WT cells and in aged primary skin fibroblasts compared to young fibroblasts. **e,** Expression *Z*-scores of 98 SRF/MRTFA/MRTFB-associated genes upregulated in mutant-expressing IMR-90 SV40-T cells compared to Empty and WT cells. **f,** Correlation between H2B turnover rates (*t*_*1/2*_, measured in Fig. 1c) and mean expression *Z*-scores of gene sets associated with SRF/MRTFA/MRTFB, TNF/NFKB, STAT3, FLT1, HIF1A, NFE2L2 and TP53 that are upregulated in mutant-expressing IMR-90 SV40-T cells compared to Empty and WT cells. Each point represents a mutant or WT condition. Linear regression lines and Pearson correlation coefficients (*r*) are shown. **g,** UMAP plot of scRNA-seq from IMR-90 SV40-T cells expressing H2B WT, E76K or E76R. **h,** Log_2_ fold-changes in cell population abundance across clusters for E76K/WT and E76R/WT comparisons in scRNA-seq analysis. **i,** GSEA of hallmark pathways enriched in each cluster compared to all other clusters in scRNA-seq analysis. See Extended Data Fig. 5e for full results. j, IPA upstream regulator analysis showing representative signaling pathways enriched in each cluster compared to all other clusters. **k,** Cluster-wise log_2_ fold-changes of inflammatory/stress-related or fibroblast identity gene expression in scRNA-seq analysis of E76R- versus WT-expressing IMR-90 SV40-T cells.

To test whether the aging-like identity and stress signatures observed in mouse tissues are recapitulated in human cells, we profiled the transcriptome of IMR-90 SV40-T human fibroblasts expressing H2B WT or mutants, alongside intact primary human dermal fibroblasts from young (ages 23–33) and aged (ages 64–72) donors. PCA clearly separated mutant-expressing cells from controls (Empty and WT), and aged from young fibroblasts (Fig. 3a,b). GSEA revealed that H2B mutants recapitulated aged fibroblast signatures, including upregulation of myogenesis, EMT, TGFb signaling, inflammatory and stress-response programs (Fig. 3c and **Extended Data Fig. 5a**). Despite these phenotypic similarities, mutant-expressing cells exhibited distinct cell-cycle and growth profiles compared to aged fibroblasts, indicating that the similarities between H2B mutants and aging are independent of proliferative state. IPA predicted activation of SRF, MRTFA/B and SMAD3—key regulators of smooth muscle identity and myofibroblast differentiation^49–51^—as exemplified by increased *ACTA2* and *ACTG2* expression (Fig. 3d,e and **Extended Data Fig. 5b**). Concurrently, proinflammatory and stress pathways, including TNF, NFKB, STAT3, AP1, MYC, FLT1, HIF1A, NFE2L2 and p53 pathways, were upregulated (Fig. 3d and **Extended Data Fig. 5c**). The magnitude of these responses increased progressively as nucleosome stability decreased, reflecting the direct impact of nucleosome instability (Fig. 3f). Together with the mouse data, these results demonstrate that nucleosome instability drives an aging-like identity and stress remodeling in human fibroblasts.

Because bulk mRNA-seq reflects averaged transcriptomic changes across heterogeneous cell populations, we next applied single-cell RNA sequencing (scRNA-seq) to resolve distinct cellular responses to nucleosome instability. UMAP analysis identified nine transcriptionally distinct clusters across WT-, E76K- and E76R-expressing cells (Fig. 3g). Mutant-expressing cells prominently expanded clusters 8 and 9 while reducing cluster 7, with E76R showing higher effects than E76K, as expected (Fig. 3h and **Extended Data Fig. 5d**). Pathway analysis of genes enriched in each cluster revealed that cluster 8 was enriched for both myogenesis and inflammatory/stress-response programs, whereas clusters 7 and 9 exhibited fewer enriched pathways (Fig. 3i and **Extended Data Fig. 5e,f**). IPA confirmed the unique concurrent activation of both stress and SRF-regulatory pathways in cluster 8, relative to other cell populations (Fig. 3j and **Extended Data Fig. 5g**). Differential expression analysis between WT and mutants in each cluster showed global upregulation of inflammatory and stress-response genes, most prominently in cluster 8 (Fig. 3k and **Extended Data Fig. 5h**). Meanwhile, SRF/MRTFA/B-associated genes such as *ACTA2* and *ACTG2* were broadly induced across clusters, accompanied by suppression of mesenchymal markers *MALAT1* and *VIM*, indicating a global basal-level shift toward a specific myofibroblast-like identity (Fig. 3k). Together, these findings demonstrate that nucleosome instability reshapes fibroblast identity by remodeling stress and myogenic programs, altering cluster composition and inducing a myofibroblast-like shift reminiscent of aged fibroblasts^52,53^.

### Nucleosome instability remodels transcriptional programs independent of changes to steady-state chromatin accessibility

To examine whether nucleosome instability alters chromatin organization, we profiled IMR-90 SV40-T cells expressing H2B WT or mutants for chromatin accessibility and transcriptional activity. MNase digestion revealed no detectable change in global chromatin accessibility (**Extended Data Fig. 6a**). To assess locus-specific effects, we performed ATAC-seq using TGFb1 treatment as a positive control (**Extended Data Fig. 6b**). E76K expression induced 204 open and 69 closed regions compared to controls, with more than half of the open regions (126/204) overlapping with TGFb-responsive sites (**Extended Data Fig. 6c**), consistent with the observed upregulation of TGFb pathway genes (Fig. 2c). Despite these modest changes, E76K-responsive sites were largely restricted to distal regulatory elements (>±5 kb, up to 100 kb from TSS), and TGFb treatment induced widespread remodeling of accessibility (>8,500 open or closed regions in both E76K and control cells) (**Extended Data Fig. 6c,d**), indicating that nucleosome instability has only minimal impact on gene-associated steady-state accessibility.

We further assessed the effect of nucleosome instability on transcriptional activity using SLAM-seq, which detects nascent transcripts (**Extended Data Fig. 6e**). SLAM-seq revealed that both myogenic and stress-related pathway genes, including myofibroblast-related genes such as *ACTA2* and *ACTG2*, were transcriptionally upregulated, consistent with bulk mRNA-seq results (**Extended Data Fig. 6f,g**). However, most transcriptional changes (115/119 upregulated and 168/170 downregulated genes) occurred independently of alterations in chromatin accessibility (**Extended Data Fig. 6h–j**). Overall, nucleosome instability reshapes transcriptional programs through enhanced nucleosome dynamics and turnover, without substantially altering global or locus-specific steady-state chromatin accessibility.

### Nucleosome instability drives premature aging

**Figure 4:**
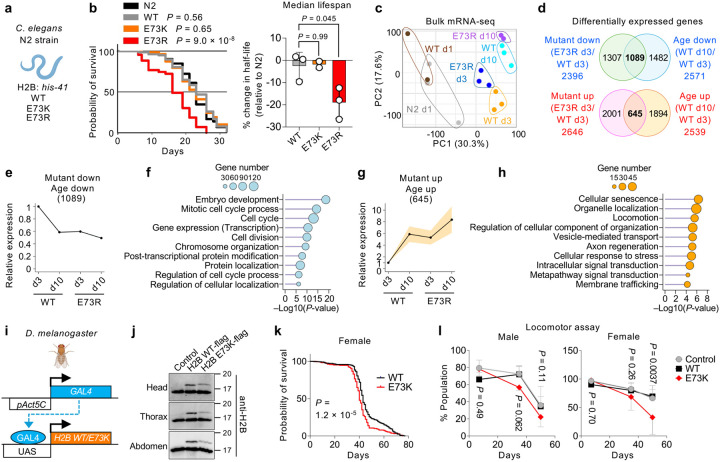
Nucleosome instability drives premature aging, reflected in transcriptional and phenotypic changes, in *C*. *elegans* and *D. melanogaster*. **a**, Schematic model of *C. elegans* expressing H2B/*his-41* WT or mutant generated via CRISPR-Cas9 in the N2 strain background. **b,** Representative Kaplan–Meier survival curves of *C. elegans* expressing H2B WT, E73K, E73R or unmodified N2 strain from three independent experiments. Log-rank tests were used to compare WT or each mutant to N2; *P*-values are indicated on the graph. Bar plot depicts median lifespan relative to N2 across three independent experiments. Statistical significance among groups was assessed by one-way ANOVA (*P* = 0.034), followed by post hoc pairwise comparisons using estimated marginal means (emmeans) with Tukey’s HSD adjustment. Only Tukey-adjusted *P*-values are shown. **c,** PCA of transcriptomic profiles from adult *C. elegans* at days 1, 3 and 10 post-adulthood. Each dot represents an individual worm (n = 3, N2 d1; 2, WT d1; 3, WT d3; 3, E73R d3; 3, WT d10; 2, E73R d10). **d,** Differentially expressed genes from mRNA-seq in *C. elegans* expressing H2B WT or E73R at days 3 or 10. **e,** Relative expression of genes downregulated both by E73R at day3 (E73R d3 vs WT d3, mutant down) and by aging in WT (WT d10 vs WT d3, age down). Shaded areas indicate 95% confidence intervals (sky blue, though nearly invisible due to low values). **f,** Gene ontology enrichment (Metascape) of 1,089 genes downregulated by both E73R and with age. g, Relative expression of genes upregulated both by E73R at day3 (E73R d3 vs WT d3, mutant up) and by aging in WT (WT d10 vs WT d3, age up). Shaded areas indicate 95% confidence intervals (orange). **h,** Gene ontology enrichment (Metascape) of 645 genes upregulated with both E73R and with age. **i,** Schematic model of the GAL4-UAS system used in *D. melanogaster* to express H2B WT or E73K under the control of the *Act5C* ubiquitous promoter. j, Western blot of H2B-Flag in dissected fly tissues. **k,** Representative Kaplan–Meier survival curves of female *D. melanogaster* expressing H2B WT or E73K from two independent experiments. A total of 365 WT and 188 E73K flies were analyzed. *P*-value was calculated using the log-rank test comparing E73K to WT. l, Locomotor performance assay of male and female *D. melanogaster* expressing H2B WT, E73K or wild-type controls. Data were analyzed by two-way ANOVA. In females, both genotype and age effects were significant (*P* = 0.030 and 1.1 × 10^−4^, respectively), whereas in males only age was significant (*P* = 7.1 × 10^−8^). Post-hoc pairwise comparisons between E73K and Control/WT at each age were calculated using emmeans with Tukey’s HSD adjustment; comparisons are shown for both sexes for consistency, and only Tukey-adjusted *P*-values are shown.

Since nucleosome instability elicits aging-like features in both tissue and cellular levels, we next examined its organismal impact in *Caenorhabditis elegans* and *Drosophila melanogaster*. Histone H2B is highly conserved among worms, flies and human, with human E76 corresponding to E73 in worms and flies (**Extended Data Fig. 7a).** Using CRISPR/Cas9, we engineered *C. elegans* strains to express E73K or E73R mutant H2B from *his-41/C50F4.5* locus^54^ (Fig. 4a and **Extended Data Fig. 7b**). Although the E73K mutant had no discernible effect, the more disruptive E73R mutant significantly shortened worm lifespan by 19% compared to N2 control (Fig. 4b). Transcriptomic profiling at post-adult day 3 revealed that E73R mutants exhibited distinct transcriptomic signatures from WT, including suppression of embryonic development and cell cycle genes, and activation of stress-response pathways (Fig. 4c and **Extended Data Fig. 7c–g**). By day 10, defense response genes, including immune components, were markedly diminished (**Extended Data Fig. 7h,i**). Of the 2,396 genes downregulated in E73R mutants at day 3, 1,089 also declined during normal aging in WT worms by day 10 (Fig. 4d,e). These genes were enriched for embryo development and cell cycle regulation (Fig. 4f), suggesting premature decline of the germline, which comprises a large proportion of total adult worm cells. Conversely, E73R upregulated 2,646 genes at day 3, including 645 genes upregulated during WT aging (Fig. 4d,g). These were enriched for pathways involving cellular senescence, organelle localization, locomotor activity and stress responses, reflecting early onset of degenerative phenotypes (Fig. 4h).

In *D. melanogaster*, H2B WT and mutant were expressed using the GAL4/UAS system under the ubiquitously active *Act5C* promoter (Fig. 4i). Western blotting confirmed expression of ectopic H2B WT and E73K across whole bodies at lower levels than endogenous H2B (Fig. 4j). Notably, E73K expression modestly reduced lifespan in female flies, but had no measurable effect in males (Fig. 4k and **Extended Data Fig. 7j**). Consistently, mutants accelerated age-associated decline in locomotor performance in females, with males also showing a subtle trend toward reduction (Fig. 4l). Collectively, these findings demonstrate that nucleosome instability promotes premature aging phenotypes, from transcriptome to function to lifespan, across evolutionarily diverse organisms.

### Nucleosome instability reduces cellular stress resilience

**Figure 5:**
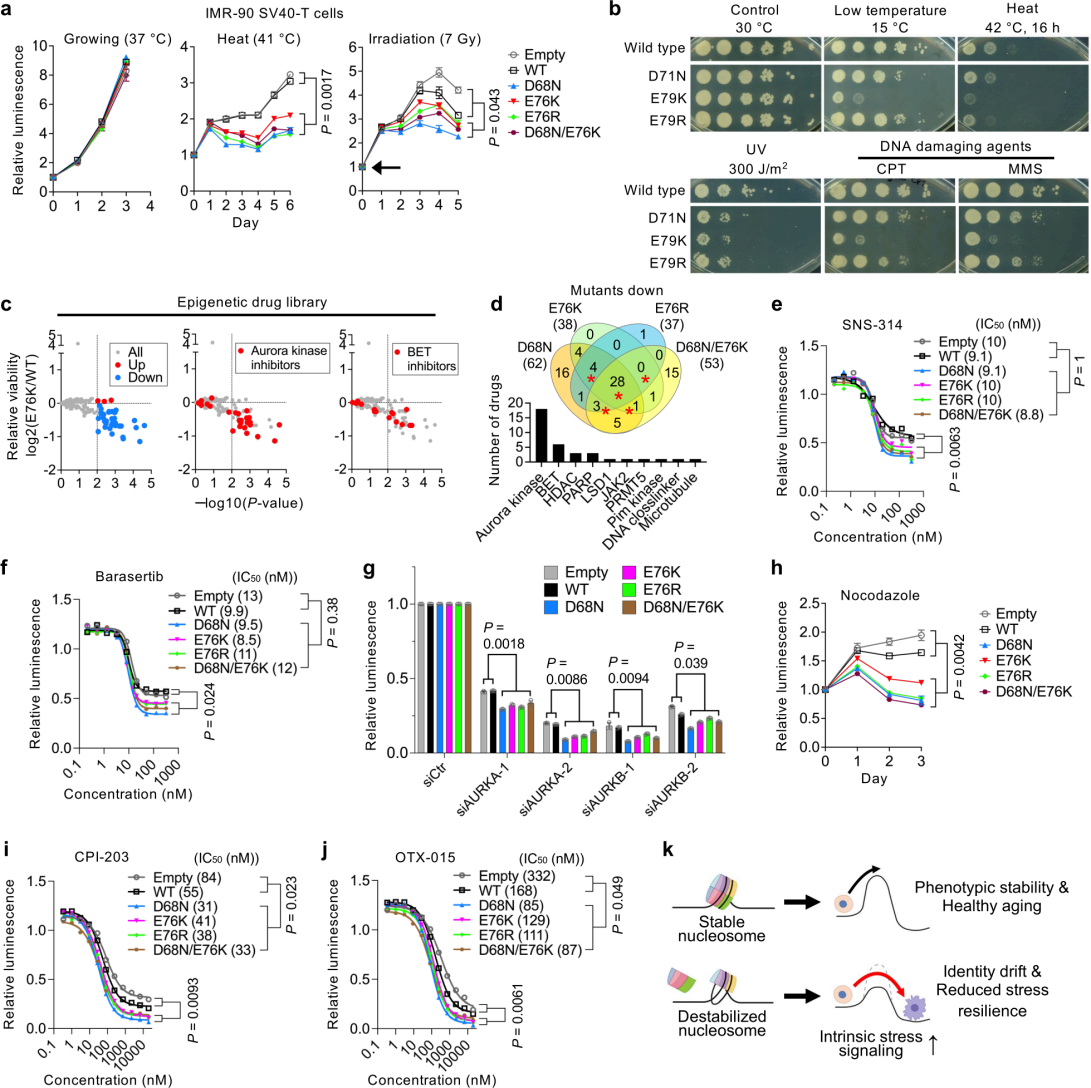
Nucleosome instability reduces cellular stress resilience across environmental and epigeneticchallenges. **a,** Cell viability assay under normal condition (37 °C), heat stress (41 °C) or after irradiation (7Gy) in IMR-90 SV40-T cells expressing H2B WT, mutants or an empty vector. Arrow indicates the day ofirradiation. Data represent mean ± s.d. from three replicates, each consisting of 4–10 wells percondition. P-values were calculated using unpaired, two-tailed Student’s t-tests comparing the means ofall mutants to Empty and WT at the final time point. Data shown are representative of two independent experiments for growing and heat stress, and similar trends were observed with two independent experiments at 5 Gy and 10 Gy irradiation (data not shown). **b,** Stress resistance assays in yeast strains expressing HTB1 WT, D71N, E79K or E79R under various stress conditions: control (30 °C for 2 days), low temperature (15 °C for 5 days), heat shock (42 °C for 16 h, then 30 °C until 2 days), UV irradiation (300 J/m^2^, then 30 °C until 2 days) and DNA damage (camptothecin (CPT) or methyl methanesulfonate (MMS) for 2 days). Cells were spotted as 1:10 serial dilutions starting from 2 OD_600_ from left to right. Experiments were performed for 3, 4 and 3 independent clones for D71N, E79K and E79R mutants, respectively, and a representative image is shown. **c,** Relative cell viability of E76K-expressing IMR-90 SV40-T cells compared to WT-expressing cells across a library of 336 epigenetic compounds. Cell viability was measured on day 4 post-treatment. Data represent the relative fold-change of the means between E76K and WT from three independent plates per condition. Similar enrichment for Aurora kinase and BET inhibitors was observed in two independent screening experiments. **d,** Venn diagram showing compounds that increased vulnerability in each mutant- versus WT-expressing cells. Bar plot highlights targets of compounds consistently reducing viability across four mutants. **e,f,i,j,** Dose–response curves for Aurora kinase inhibitors (SNS-314 mesylate and barasertib) and BET inhibitor (CPI-203 and OTX-015) in IMR-90 SV40-T cells expressing H2B WT, mutants or empty vector, with corresponding IC_50_ values. Data represent means from three replicates, each consisting of six wells per condition. *P*-values were calculated using unpaired, two-tailed Student’s *t*-tests on IC_50_ values and on the means of all mutants compared to controls at the bottom plateau from nonlinear regression. **g,** Cell viability after three days of siRNA treatment targeting *AURKA* or *AURKB* in IMR-90 SV40-T cells expressing H2B WT, mutants or empty vector. Data represent mean ± s.d. from three replicates, each consisting of 14 wells per condition. *P*-values were calculated using unpaired, two-tailed Student’s *t*-tests comparing the means of all mutants to controls. **h,** Cell viability assay with 0.1 mM nocodazole in IMR-90 SV40-T cells expressing H2B WT, mutants or empty vector. Data represent mean ± s.d. from three replicates, each consisting of 16 wells per condition. *P*-values were calculated using unpaired, two-tailed Student’s *t*-tests comparing the means of all mutants to controls at the final time point. Data are representative of two independent experiments. **k,** Schematic model illustrating how nucleosome stability influences cellular outcomes. Stable nucleosomes maintain phenotypic stability and support healthy aging. Destabilized nucleosomes disrupt metastable lineage programs with compromised homeostatic stress signaling, leading to identity drift and reduced stress resilience.

Given that nucleosome instability consistently activated stress-response genes across multiple models, we reasoned that it might compromise cellular stress homeostasis and reduce stress resilience. Indeed, IMR-90 SV40-T cells expressing H2B mutants showed reduced viability following heat stress or irradiation (Fig. 5a). To assess evolutionary conservation, we tested stress tolerance in *Saccharomyces cerevisiae*. Yeast possess two H2B genes (*HTB1* and *HTB2*); we replaced *HTB1* with mutants equivalent to human D68N, E76K and E76R (*HTB1* D71N, E79K or E79R) while depleting *HTB2* (**Extended Data Fig. 8a,b**). As in human fibroblasts, these mutants did not impair proliferation (**Extended Data Fig. 8c**) or replicative lifespan (**Extended Data Fig. 8d**), but markedly reduced tolerance to various stressors, including cold, heat, UV and genotoxic agents such as camptothecin (CPT) and methyl methanesulfonate (MMS) (Fig. 5b).

To assess cellular resilience to chromatin and epigenetic perturbations, we screened 336 compounds targeting epigenetic regulators using IMR-90 SV40-T cells expressing H2B mutants. Mutant-expressing cells were generally more sensitive, showing pronounced vulnerability to Aurora kinase inhibitors and bromodomain and extraterminal (BET) inhibitors (Fig. 5c,d and **Extended Data Fig. 8e**). For Aurora kinase inhibitors (SNS-314, Barasertib and GSK1070916), drug tolerance assays confirmed reduced tolerance, although IC_50_ values remained unchanged (Fig. 5e,f and **Extended Data Fig. 8f**). Knockdown of *AURKA* or *AURKB* phenocopied this reduced tolerance (Fig. 5g). Aurora kinases play pivotal roles in mitotic progression^55^. Indeed, mutant-expressing cells exhibited modest G2/M-phase and polyploid cell accumulation, which was further exacerbated under stress (**Extended Data Fig. 8g,h**), and showed decreased tolerance to mitotic inhibitors such as nocodazole and paclitaxel (Fig. 5h and **Extended Data Fig. 8i**). In contrast, for BET inhibitors (CPI-203, OTX-015 and (+)-JQ-1), both tolerance and IC_50_ were reduced (Fig. 5i,j and **Extended Data Fig. 8j**), indicating increased sensitivity to epigenetic perturbation. Together, these findings demonstrate that nucleosome instability compromises cellular stress resilience, in part through reduced epigenomic integrity and increased mitotic vulnerability.

## Discussion

Our findings establish that, as predicted by the ITOA, nucleosome stability acts as an essential epigenomic safeguard that preserves cell identity. In addition, we find that nucleosome stability supports lineage specification, maintains stress resilience and sustains healthy tissue function throughout life. Although nucleosome dynamics are regulated by histone variants, histone chaperones and chromatin remodelers and modifiers^15,16^, it has remained untested whether perturbing intrinsic nucleosome stability influences the fundamental cellular and tissue properties that shape aging. We show that reduced nucleosome stability is sufficient to destabilize cell identity, alter lineage-specific gene programs and impair cellular stress responses in a manner reminiscent of aging—without broadly affecting steady-state chromatin accessibility, DNA damage, proliferation or viability. These results challenge and extend the conventional view that loss of cell identity arises from cell cycle arrest, persistent DNA damage, global chromatin remodeling, senescence or niche alterations^3,56–60^. Instead, nucleosome stability itself emerges as an intrinsic mechanism that safeguards cell identity and lineage fidelity.

Nucleosome instability compromised cell identity gene expression programs reminiscent of aging across diverse cell and tissue models. In skeletal muscle, it reduced fast-twitch myogenesis^61^; in heart, impaired fatty acid metabolism^62^; in HSPCs, biased transcription toward megakaryocyte/erythroid lineages^36–39^; and in human fibroblasts, induced a myofibroblast-like mesenchymal drift^52,63^. These cell type-specific alterations suggest that certain lineage programs reside in “metastable” states that are normally stabilized by nucleosome integrity. Loss of this stabilizing barrier amplifies fluctuations within these states, driving transcriptional drift and cell identity loss (Fig. 5k). Consistently, scRNA-seq in human fibroblasts revealed expansion of a cluster enriched for stress and myofibroblast-like programs, indicative of cell-state transitions from a metastable fibroblast identity. This framework aligns with previous studies linking chromatin dynamics to transcriptional noise^64^, cell fate metastability^65^ and age-associated transcriptional drift^66^. Our findings extend these concepts by showing that intrinsic nucleosome stability contributes to maintaining metastable lineage programs and thereby preserving cell identity.

Nucleosome instability activated intrinsic inflammatory and stress pathways, including TNF–NF-kB, interferon and p53 signaling, across multiple models. In human fibroblasts, these responses occurred without broad genotoxicity or global/local changes in chromatin accessibility, indicating that nucleosome instability itself constitutes an intrinsic form of stress, rather than reflecting canonical damage pathways such as persistent DNA lesions^60^. These findings reveal a previously underappreciated role for nucleosome stability in maintaining homeostatic stress signaling. Across human cells and yeast, nucleosome instability reduced cellular resilience to exogenous challenges, including environmental, epigenetic and mitotic stressors. Specifically, cells showed reduced tolerance to Aurora kinase inhibitors, reflecting mitotic vulnerability, and increased sensitivity to BET inhibitors, indicating heightened dependence on BET-regulated transcriptional programs^67^. Collectively, these results demonstrate that nucleosome instability functions as an intrinsic form of stress that undermines epigenomic integrity and compromises cellular stress resilience (Fig. 5k).

The impact of nucleosome instability extends from the cellular to the organismal level. In worms and flies, destabilizing nucleosomes shortened lifespan and accelerated the onset of age-associated transcriptomic and behavioral deficits. These observations suggest that nucleosome stability serves as an evolutionarily conserved safeguard whose loss compromises cellular resilience and accelerates organismal aging.

Mutations at the H2B–H4 interface have been identified in various cancers^18–20^, yet their functional impact remains poorly understood. Given that both altered cell identity and dysregulated stress signaling are hallmarks of cancer cells^68^, our data suggest that nucleosome instability may prime cells for malignant transformation by weakening tumor-suppressive cell identity maintenance programs while activating pro-tumorigenic inflammatory and stress signaling pathways. This framework unifies cell identity drift and stress signaling under a common epigenetic dysregulation that could predispose cells to cancer. Further studies are needed to elucidate the causal relationship between nucleosome instability and tumorigenesis.

A key limitation of this study is that the impact of nucleosome instability varies across tissues, histone residues and model organisms. For instance, the liver exhibited relatively modest effects compared to skeletal muscle and heart. In worms, the E73R mutant shortened lifespan, whereas the weaker E73K mutant had no measurable effect. Moreover, because histones are abundant proteins encoded by more than ten genes per genome in human, mouse, fly and worm, our experimental systems replaced less than 50% of endogenous histones with mutant variants. Therefore, both the quantitative extent of histone replacement and the degree of nucleosome destabilization likely contribute to the observed phenotypic variability. We also note that, although our data support the ITOA, they do not imply that the epigenome is the sole determinant of the pace of aging. Rather, we posit that the many molecular, cellular and systemic processes causally implicated in aging act within a homeostatic network, such that disruption of any one of these “hallmarks” can destabilize the network and accelerate aging.

Future work should define the molecular determinants of nucleosome stability and its homeostasis; examine how susceptibility to nucleosome destabilization varies across cell types and tissues and by the degree of nucleosome destabilization; determine whether nucleosome perturbations contribute causally to aging within a broader homeostatic network; and explore whether stabilizing nucleosome conformations can counteract aging and age-associated diseases, including cancer. Addressing these questions could establish whether nucleosome stability within the framework of ITOA is a tractable target for interventions to extend healthspan and reduce disease risk.

## Methods

### Plasmid preparation

Point mutations in human histone H2B genes (coding region of *H2BC5* (NM_138720.2)) (WT, D68N, E76K, E76R, D68N/E76K) were cloned into pLenti CMV EGFP puro vector (addgene #17448) at BamHI-SalI site, which removed EGFP from the plasmid, with either C-terminal Flag tag. For EGFP or SNAP tag vectors, H2B or H2A (coding region of *H2AC4* (AK311785.1)) was cloned into pLenti PGK EGFP puro vector at BamHI-SalI site, where the CMV promoter was replaced by human PGK promoter from pLKO.1-blast (addgene #26655) at ClaI-BamHI site. EGFP/SNAP was fused C-terminally to H2B and N-terminally to H2A. Simian virus 40 large T antigen was cloned from pBABE SV40 zeocin T-antigen ER (kindly provided from Robert Weinberg) at BamHI site and inserted into pLenti PGK EGFP blast vector, where puromycin resistant gene was replaced by blasticidin resistant gene from pLKO.1-blast. For transduction of mouse HSPCs, mouse H2B genes (coding region of H2bc4 (NM_023422.3)) (WT, D68N, E76K, E76R) were cloned into MSCV PIG (Puro IRES GFP) (kindly provided by Aniruddha Deshpande, addgene #18751) at BglII-XhoI site with C-terminal Flag tag. For transduction of mouse tissues, mouse H2B (coding region of *H2bc4* (NM_023422.3)) (WT, D68N, E76K, E76R) were cloned into single-stranded AAV vector (VectorBuilder) with C-terminal Flag tag under the control of CMV promoter and Woodchuck hepatitis virus posttranscriptional regulatory element (WPRE). All constructs were verified by Sanger sequencing.

### Viral preparation

Lentiviruses were generated from 293 T cells transfected with expression vector, VSV-G envelope vector and psPAX2 packaging vector using lipofectamine 2000 (Invitrogen 52887). For retrovirus preparation, 293 T cells were transfected with expression vector and Ecopack gag/pol/env plasmid (a gift from Aniruddha Deshpande) using lipofectamine 2000. The virus supernatant was collected over 2 days, filtered through 0.45 mm membrane and directly used for the experiment.

Large-scale rAAV productions were carried out by the SBP Functional Genomics Core Facility. Briefly, HEK-293T cells were screened and optimized to establish high-titer, virus-producing clonal lines using Biomek i7 automation (Beckman Coulter). For production at scale, twenty 150 cm^2^ plates of low-passage, freshly cultured clonal 293T cells were co-transfected with rAAV vector, RepCap9 (a gift from James M. Wilson; addgene, 112865) and Helper (Cell Biolabs) plasmid DNA using polyethylenimine (Polysciences). At 84 h post-transfection, crude viral particles were harvested from the supernatant by polyethylene glycol precipitation and from the cell pellet through sonication, followed by benzonase (MilliporeSigma) treatment. Unconcentrated viruses were then purified by iodixanol (MilliporeSigma) gradient ultracentrifugation. The fraction containing concentrated rAAV particles was collected, buffer-exchanged into PBS, aliquoted and frozen at −80°C for long-term storage. Viral genome titers were measured via SYBR Green-based qPCR (Roche Diagnostics).

### Cell culture

IMR-90 SV40-T cells were maintained in Dulbecco’s modified Eagle’s medium (Gibco, 10313–121) supplemented with 10% fetal bovine serum (Corning, 35–011-CV), 2 mM L-glutamine, and 1% penicillin/streptomycin (Gibco, 15140–122). Cells were cultured at 37 °C in 5% CO_2_ and 5% O_2_. Stable cell lines expressing wild-type (WT) or mutant (D68N, E76K, E76R, D68N/E76K) were generated via lentiviral transduction followed by selection with puromycin (1 μg/ml) for two days.

For stress resilience assays, cells were either cultured continuously at 40 °C in 5% CO_2_ and 5% O_2_ or exposed once to ionizing radiation at a dose of 7 Gy (Rad Source RS-2000), followed by continued culture under the same conditions. For gene knockdown experiments, cells were transfected using Lipofectamine RNAiMAX (Thermo Fisher Scientific) with siRNAs targeting AURKA (siAURKA-1 (Invitrogen, s196); siAURKA-2 (Invitrogen, s197)) and AURKB (siAURKB-1 (Invitrogen, s17611); siAURKB-2 (Invitrogen, s17612)) or a non-targeting control (siCtr (Dharmacon, D-001810-01-05)) at a final concentration of 5 nM. Transfected cells were incubated for three days prior to viability assessment using CellTiter-Glo (Promega), with luminescence detected on an EnVision 2103 multilabel reader (PerkinElmer). All stress assays and siRNA treatments were performed in 384-well plates (Greiner Bio-One 781098). Cells and reagents were dispensed using a Multidrop Combi reagent dispenser (Thermo Fisher Scientific).

As a positive control for double-stranded DNA breaks, cells were treated with etoposide at a final concentration of 10 mM for 24 h prior to fixation.

### Drug tolerance assay

Cells were seeded into 384-well plates and treated with increasing concentrations of Aurora kinase inhibitors (SNS-314 Mesylate (SelleckChem, S8699); Barasertib (SelleckChem, S1147); GSK1070916 (MedChemExpress, HY-70044)) and BET inhibitors (CPI-203 (Sigma, SML1212); OTX-015 (Sigma, SML1605); (+)-JQ-1 (MedChemExpress, HY-13030)). Cells were treated for three days for Aurora kinase inhibitors and four days for BET inhibitors prior to viability assays. Paclitaxel (Cayman Chemical, 10461) and nocodazole (MedChemExpress, HY-13520) were also used in selected assays. After drug treatment, cell viability was measured using CellTiter-Glo, and luminescence was detected with an EnVision 2103 multilabel reader. Drug sensitivity was assessed using GraphPad Prism by calculating IC_50_ values from nonlinear regression of cell viability curves, while drug tolerance was quantified as the fitted bottom asymptote, representing residual viability at high drug concentrations. Sensitivity to nocodazole and paclitaxel was evaluated over a multi-day time course by monitoring luminescence-based viability.

### Epigenetic library screen

A high-throughput screen of an epigenetic drug library was performed to identify compounds that selectively impaired viability in mutant cell lines. The library was provided by the SBP Conrad Prebys Center for Chemical Genomics. Compounds from the Epigenetics compound library and DMSO controls were transferred into 384-well plates using an Echo 655 Liquid Handler (Beckman Coulter). The drug concentration was 1 mM (2.5 nL of 10 mM drugs). Cells were incubated for four days, and viability was measured using CellTiter-Glo with an EnVision 2103 multilabel reader. Relative viability was calculated following drug treatment and plotted against statistical significance (−log_10_ P-value). Comparative analysis across mutants was visualized using Venn diagrams and bar graphs to identify overlapping drug sensitivities.

### Cell cycle assay

Cell cycle distribution was assessed using the Click-iT Plus EdU Pacific Blue Flow Cytometry Assay Kit (Invitrogen, C10636), following the manufacturer’s instructions. Cells were subjected to either heat stress (40 °C for 24 h) or irradiation (7 Gy) 24 h prior to the experiment. EdU incorporation was used to label cells undergoing DNA synthesis, and total DNA content was counterstained with propidium iodide (PI; Invitrogen, F10797). Flow cytometry was performed on a Novocyte flow cytometer (ACEA Biosciences Inc.), and data were analyzed using FlowJo software. Cell cycle phases (G1, S, G2/M) were determined by EdU incorporation and PI intensity. Populations with DNA content greater than 4N were also quantified to assess potential re-replication or polyploidy.

### Sequential salt fractionation

Sequential salt fractionation was performed, as previously described^69^. Briefly, 3 × 10^6^ IMR-90 SV40-T cells expressing WT or mutant H2B were lysed in 100 ml of modified buffer A (25 mM HEPES pH 7.6, 25 mM KCl, 5 mM MgCl_2_, 0.05 mM EDTA, 0.1% NP-40, 10% glycerol) on ice. Lysates were centrifuged, and the supernatant was collected as the cytoplasmic fraction. Pellets were washed once with 0 mM NaCl mRIPA (50 mM Tris-HCl pH 8.0, 1% NP-40, 0.25% sodium deoxycholate), then sequentially resuspended in mRIPA containing 0, 0.6, 0.9 or 1.2 M NaCl and centrifuged at 12,000 × *g* for 5 min. Nuclear extracts were collected at each step. Samples were resuspended in Laemmli sample buffer and used for western blotting.

### Fluorescence recovery after photobleaching (FRAP)

IMR-90 SV40-T cells expressing H2B-EGFP constructs were seeded on PhenoPlate^™^ 96-well microplates (PerkinElmer) one day prior to the experiment. EGFP fluorescence was imaged using a Nikon N-SIM E super-resolution/A1 ER confocal microscope with a 60 × oil immersion objective under temperature control (37 °C). A rectangular region of interest, approximately half of the nucleus, was bleached with the 488-nm laser at full power for 45 s, and recovery was monitored at 4 min intervals for 60 min. Fluorescence intensities at the bleached site were corrected using the unbleached region of the same nucleus. Recovery curves were fitted with a single-exponential model in GraphPad Prism to derive plateau values (*Ymax*). More than fifteen nuclei were analyzed per condition.

### Histone turnover assay

IMR-90 SV40-T cells expressing H2B-SNAP constructs were labeled with 1.5 mM SNAP-Cell TMR-Star (New England Biolabs #S9105) in DMEM for 30 min in the incubator, washed with PBS, and blocked with 5 mM SNAP-Cell Block (New England Biolabs #S9106) in DMEM. After a 0–30 h chase period, residual nuclear fluorescence was imaged on a Nikon T2 microscope with automated image capture. Cells were then fixed and counterstained with DAPI. Images were analyzed in NIS Elements AR v5.21.03 using dark background subtraction, thresholding, size exclusion and automated partitioning to identify nuclear DAPI features. At least 2,600 nuclei were analyzed per condition. Signal intensities were normalized to time 0 and fitted to a one-phase exponential decay model in GraphPad Prism. The degradation rate constant (*k*) was obtained from the fit, and the protein half-life (*t*_*1/2*_) was calculated as ln2/*k*. To ensure accurate modeling of the decay, the plateau parameter was constrained to zero during curve fitting.

### Mouse models

Male 3-week-old C57BL/6J mice (The Jackson Laboratory, strain #000664) were used. A total of 100 ml of rAAVs (1–5 × 10^12^ VG) were administered via retro-orbital injection by Sanford Burnham Prebys Medical Discovery Institute Animal Facility. Mice were housed five per cage and maintained under controlled temperature (22.5 °C) and a 12 h light/dark cycle, with food and water provided *ad libitum*. Four weeks post-transduction, mice were euthanized with CO_2_, and then quadriceps skeletal muscle, heart and liver were collected, snap-frozen in liquid nitrogen and stored at −80 °C. All animal procedures were approved by the Institutional Animal Care and Use Committee (IACUC) of Sanford Burnham Prebys Medical Discovery Institute, and all experiments were performed at Sanford Burnham Prebys Medical Discovery Institute Animal Facility in compliance with the IACUC guidelines and relevant ethical regulations for animal research.

### Histology and H&E staining

Tissues were paraffin-embedded, sectioned at 5 μm, and mounted on glass slides. Slides were dried at 58 °C for 1 h, deparaffinized, and rehydrated through graded alcohols. Hematoxylin and eosin (H&E) staining was performed using a Leica Autostainer XL (Leica Microsystems) following standard protocols. All histological processing and staining were carried out by the Histology Core Facility at Sanford Burnham Prebys Medical Discovery Institute.

### HSPCs isolation from mouse bone marrow

Bone marrow cells were isolated from spinal cord and hind leg bones of 6 male, 9-week-old C57BL/6 mice. Collected bones were placed in 15% FBS/PBS, then crushed using a pestle in a mortar and filtered through a 40 mm cell strainer (Fisher Scientific). Cells were pelleted by centrifugation, resuspended in lysis buffer (BD Biosciences, 555899) and incubated for 4 min to lyse red blood cells. After washing twice with 15% FBS/PBS, lineage-positive cells were depleted using the EasySep Mouse Hematopoietic Progenitor Cell Isolation Kit (STEMCELL, #19856). Briefly, mouse FcR blocker was added to the sample, followed by the hematopoietic progenitor cell isolation cocktail and incubated for 15 min at 4 °C. Streptavidin RapidSpheres were then added and incubated for 10 min before placing the sample in the magnet; the supernatant containing lineage-negative cells was collected. Cells were pelleted, resuspended at 20 × 10^6^ cells/ml in PBS containing 2% FBS and 1 mM EDTA, and subjected to flow cytometric sorting.

Sorting was performed using a FACSAria III cell sorter (BD Biosciences, P0024) with FACSDiva software v8.0.1. Biotin-positive cells were excluded by gating with a BV421 fluorescence minus one (FMO) control [405/450 50-A], and subsequent gates were set using PE and APC FMOs to define Sca-1^+^ and c-Kit^+^ populations, respectively. FMOs were used to establish gating thresholds and discriminate true positive signals from background in populations with heterogeneous or dim marker expression. Lin^−^Sca-1^+^c-Kit^+^ (LSK) cells were collected, yielding 29,510 cells from a total of 35,115,973 bone marrow cells. Antibodies used were PE anti-mouse Ly-6A/E (Sca-1) (rat monoclonal antibody, BioLegend, #108107), APC anti-mouse CD117 (c-Kit) (rat monoclonal antibody, BioLegend, #105811), and Brilliant Violet 421 Streptavidin (BioLegend). For fluorescence compensation, single-stained controls for PE, APC, and BV421, along with an unstained control, were prepared prior to sample acquisition and used to generate a compensation matrix.

### Myeloid and megakaryocyte/erythroid lineage differentiation

HSPCs were cultured in DMEM supplemented with 15% FBS, 2 mM L-glutamine, and 1% penicillin/streptomycin, 20 ng/ml mouse SCF (Thermo Fisher Scientific, 250–03), 10 ng/ml mouse IL-6 (Thermo Fisher Scientific, 216–16) and 6 ng/ml mouse IL-3 (Thermo Fisher Scientific, 213–13). One day post-isolation, cells were transduced with MSCV retroviruses for 1 day on a retronectin-coated plate, followed by puromycin selection (2.5 mg/ml) for additional 2 days. Colony-forming unit assays were performed using MethoCult GF M3434 (STEMCELL Technologies), according to manufacturer’s instructions. Briefly, approximately 5,500 cells were mixed with 4 ml of methylcellulose media supplemented with 20 ng/ml mouse FLT3L (Thermo Fisher Scientific, 250–31L) and 50 ng/ml mouse TPO (Thermo Fisher Scientific, 315–14), and plated in triplicate (1.1 mL per 3.5 mm dish) using a 16-gauge blunt-end needle (STEMCELL Technologies, 28110). Colonies were manually counted under bright-field microscopy and classified as CFU-G, CFU-M, or CFU-GM based on morphology. Cells were harvested from methylcellulose cultures 9 days after differentiation using DPBS and stored at −80 °C for mRNA-seq or used for flow cytometry. To assess differentiation efficiency, cells were stained with antibodies against lineage markers, Sca-1 (BioLegend, 108119), c-Kit (BioLegend, 105811), Gr-1 (BioLegend, 108433) and Mac-1 (BioLegend, 101212). Flow cytometry was performed, and data were using a Novocyte (ACEA Biosciences Inc.), analyzed with FlowJo software. Quantification of Sca-1^+^c-Kit^+^, Gr-1^+^ and Mac-1^+^ populations was used to assess differentiation efficiency.

### Western blotting

For protein expression analysis, cell suspensions were directly lysed in 2 × Laemmli sample buffer (125 mM Tris-HCl pH 6.8, 4% SDS, 20% glycerol, 0.2% bromophenol blue). Mouse tissues and Drosophila samples were homogenized using tissue grinding tubes (Precellys, P000917-LYSK0-A for skeletal muscle and heart; P000912-LYSK0-A for liver and flies) with a Precellys Evolution homogenizer (Bertin Technologies) for three cycles of 4 × 30 s at 6,000 × *g*. Lysates were centrifuged at 12,000 rpm for 5 min, and the supernatant was boiled at 95 °C for up to 5 min and stored at −80 °C. Protein samples (5–50 mg per lane) were separated by SDS–PAGE using 15% Criterion Tris-HCl Protein Gels (Bio-Rad, 3450020) and Tris/Glycine/SDS buffer (Bio-Rad, 1610772) and transferred to nitrocellulose membranes (Bio-Rad, 1620112) using the Trans-Blot Turbo system (Bio-Rad). Membranes were block with 5% skim milk and incubated with primary antibodies overnight at 4 °C, followed by HRP-conjugated secondary antibodies for 1 h at room temperature. Bands were visualized using enhanced chemiluminescence reagents (Thermo Fisher Scientific, 34580 or 34095) and imaged with the ChemiDoc Imaging System (Bio-Rad).

### Immunofluorescence

Cells were seeded on PhenoPlate 96-well microplates (PerkinElmer) and fixed with 10% neutral buffered formalin (Epredia, 9400–1). Fixed cells were permeabilized with buffer (0.2% Triton X-100 and 0.5% BSA in PBS) for 10 min, blocked with 0.5% BSA in PBS and incubated with primary antibodies diluted in 0.2% BSA in PBS for 1 h at room temperature. After two washes with 0.2% BSA in PBS, samples were incubated with secondary antibodies for 1 h. Images were acquired using a Nikon T2 microscope with manual or automated image capture. Image analysis was performed in NIS Elements AR v5.21.03 using dark background subtraction, thresholding, size exclusion and automated partitioning to identify nuclear DAPI features. Images were processed using Fiji^70^.

### Antibodies

For western blotting, the following primary antibodies were used: Flag M2 (Sigma, F3165, 1:5000; Cell Signaling, 14793, 1:5000), H2B (Cell signaling, 12364, 1:5000), GFP (Abcam, 6556, 1:1000) and GAPDH (Santa Cruz, sc-47724, 1:10000; Abcam, 9485, 1:10000). For immunofluorescence, the following primary antibodies were used: Flag M2 (Sigma, F3165, 1:1000; Cell Signaling, 14793, 1:1000), Phospho-Histone H2A.X (Ser139) (Millipore, 05–636, 1:1000) and Histone H2A.X (Abcam, ab11175, 1:1000). The following secondary antibodies were used: Goat anti-Mouse IgG, IgM (H+L) HRP (Thermo Fisher Scientific, 31446), Goat anti-Rabbit IgG, (H+L) HRP (Millipore, AP307P), Goat anti-Mouse IgG (H+L), Alexa Fluor 488 (Thermo Fisher Scientific, A11029), Goat anti-Rabbit IgG (H+L) and Alexa Fluor 594 (Thermo Fisher Scientific, A11012).

### RNA extraction, cDNA synthesis and qPCR

Total RNA from human IMR-90 SV40-T cells and mouse HSPCs was extracted using the Quick-RNA Miniprep Kit (Zymo Research, R1055), according to the manufacturer’s instructions. Eluted RNA was quantified using a Nanodrop 2000 spectrophotometer (Thermo Fisher Scientific) and reverse-transcribed into cDNA using the SuperScript III First-Strand Synthesis System (Invitrogen, 18080093) following the manufacturer’s instructions. qPCR was performed using SYBR green master mix (Applied Biosystems, A25742) in 384-well plates. Cycling conditions were 95 °C for 20 s, followed by 40 cycles of 95 °C for 1 s and 60 °C for 20 s, with a melt-curve analysis. The following primers were used: H2B-F (ACCAAGGCCGTCACCAAGTAC) and Flag-R (CTTGTCGTCATCGTCTTTGTAGTCTC).

For mouse tissues, frozen samples (≤0.5 × 0.5 × 0.5 mm) were placed in tissue grinding tubes (Precellys, P000917-LYSK0-A for skeletal muscle and heart; P000912-LYSK0-A for liver) containing TRIzol Reagent (Invitrogen, 15596026) and homogenized in a Precellys Evolution homogenizer (Bertin Technologies) for three cycles of 4 × 30 s at 6,000 *g*. Lysates were centrifuged at 12,000 rpm for 1 min, and the supernatant was collected. RNA was then extracted using the Direct-zol RNA Miniprep Kit (Zymo Research, R2052), according to the manufacturer’s instructions.

### RNA-seq and data analysis

For mouse and worm samples, PolyA RNA isolation and library preparation was performed with the Watchmaker mRNA Library Prep Kit (Watchmaker Genomics: 7BK0001) with xGEN Stubby adaptors (IDT, 336338420) and xGEN 10nt UDIs (IDT, 336338436). Libraries were sequenced with the Element Biosciences AVITI Sequencing platform using the AVITI 2×75 High Output Cloudbreak Freestyle Kit (Element Biosciences, 860–00015). For human IMR-90 SV40-T cell samples, PolyA RNA isolation and library preparation was performed with NEBNext Ultra II DNA Library Prep Kit for Illumina (New England Biolabs, E7645) with NEBNext Multiplex Oligos for Illumina (96 Index Primers) (New England Biolabs, E6609). Libraries were sequenced with Novaseq6000 at PE100 at the UC San Diego IGM Genomics Center.

Reads were trimmed for adaptors and low-quality bases (Q<20) using Trimmomatic (Galaxy v0.38.0) or Trim Galore! (Galaxy v0.6.7+galaxy0 or v0.6.7+galaxy1) and then aligned to the human (hg38), mouse (mm10) or worm (ce10) reference genome using HISAT2 (Galaxy v2.2.1+galaxy1). Aligned reads were quantified with featureCounts (Galaxy v2.0.3+galaxy1 or v2.0.3+galaxy2) using exon regions from gene annotation files (human: GENECODE v32; mouse: UCSC mm10; worm: NCBI Refseq ce11). Differential expression analysis was performed with DESeq2 (Galaxy v2.11.40.7+galaxy2 or v2.11.40.8+galaxy0) using the local fitting strategy.

Principal component analysis (PCA) was performed in R (v4.4.2) using the prcomp function on log_2_-transformed, normalized count data. Genes with zero variance across samples were excluded prior to PCA. The first two principal components (PC1 and PC2) were visualized using the ggplot2 package (v3.5.2), with genotype-based color coding and axes indicating the proportion of variance explained. Hierarchical clustering of differentially expressed genes (DEGs) was performed using Euclidean distance and complete linkage via the pheatmap package (v1.0.12), with rows (genes) clustered, columns (samples) fixed and *Z*-scores capped to limit extreme values. Pathway enrichment analysis was performed for hallmark and cell type signature gene sets, including Tabula Muris Senis gene sets, using GSEA 4.4.0 (Broad Institute). Upstream regulator analysis was performed using Ingenuity Pathway Analysis (IPA; QIAGEN). GSEA results were visualized as bubble plots: for hallmark signatures, point size represented −log10(FDR) and color indicated normalized enrichment score (NES); for cell type signatures, NES was plotted on the x-axis, cell type on the y-axis, point size represented −log10(FDR) and color indicated gene set size. For heatmap analysis, gene expression values were log_2_-transformed and standardized to *Z*-scores on a per-gene basis, and visualized using the ComplexHeatmap package (v2.22.0) with color scaling from circlize (v0.4.16), clustering of rows (genes) and a fixed column (samples) order. To compare gene groups, *Z*-scores of individual genes were averaged per sample to obtain mean *Z*-scores. Pearson correlation coefficients were calculated between these mean *Z*-scores and corresponding protein turnover values. Data preprocessing and manipulation were performed using dplyr (v1.1.4) and readr (v2.1.5). For immune signature analysis, bulk RNA-seq data were processed using CIBERSORTx with the LM22 signature matrix. Immune cell proportions were compared between control samples (Empty and WT) and mutant samples (D68N, E76K, and E76R) using Wilcoxon rank-sum tests. Results were visualized using boxplots with overlaid jittered points for each cell type.

### RNA-seq in primary human dermal fibroblasts

Primary human dermal fibroblasts from four young donors (age 23–33) and five aged donors (age 64–72) were obtained from the San Diego Nathan Shock Center (SD-NSC) Human Cell Models of Aging Core (see **Supplementary Table 1**). Cells were cultured in DMEM (Gibco, 11965092) supplemented with 10% fetal bovine serum (Corning, 45000–736), 1% non-essential amino acids (Gibco, 11140050) and 1% GlutaMAX (Gibco, 35050061) at 37 °C, 5% CO_2_ and ambient O_2_. For RNA-seq, 200,000 cells per cell line were seeded in single wells of 6-well plates and cultured for 48 h. RNA was extracted using the RNeasy Mini Kit (Qiagen, 74104), according to the manufacturer’s instructions. Briefly, cells were washed with Dulbecco’s phosphate-buffered saline (DPBS; Gibco, 14190094) and lysed in 350 mL RNeasy lysis buffer containing b-mercaptoethanol. Lysates were homogenized using QIAshredder columns (Qiagen, 79656), followed by purification with RNeasy Mini spin columns, including on-column DNase digestion. PolyA library prep was performed by the Salk Institute for Biological Studies Next Generation Sequencing and Genomics Core, and sequencing was carried out on an Illumina NovaSeq 6000 SP platform. Raw FASTQ files were mapped to hg19 (https://www.ncbi.nlm.nih.gov/grc/human) by STAR (v2.5.3a), and DEG analysis was performed with HOMER pipeline (v4.11.1).

### Single-cell RNA sequencing

IMR-90 fibroblasts expressing WT or mutant H2B were processed using the Chromium platform at the Center for Epigenomics at UC San Diego. Sorted cells were centrifuged, resuspended in cell media, and counted using Trypan Blue prior to loading 18,000 cells per lane onto a Chromium Controller. Libraries were generated using the Single Cell 3’ v3.1 kit (10x Genomics, 1000268) and Dual Index Kit TT Set A (10x Genomics, 1000215) according to the manufacturer’s instructions. cDNA was amplified for 12 PCR cycles, size-selected using SPRISelect (Beckman Coulter), and subjected to 10 cycles of index PCR. Library concentration was assessed with a Qubit dsDNA HS Assay Kit (Thermo Fisher Scientific). Sequencing was performed on a NextSeq500 or NovaSeq6000 (Illumina) with read lengths of 28 bp (Read 1), 91 bp (Read 2), and 10 bp for each index.

Data were processed with the Cell Ranger pipeline (v8.0.0, 10x Genomics) for demultiplexing, alignment to the GRCh38 reference genome and generation of gene–barcode count matrices. Expression matrices were analyzed using Scanpy (v1.9.8). Cells with fewer than 200 detected genes, more than 5% mitochondrial gene expression, or more than 8,000 detected genes were excluded. Genes detected in fewer than three cells were also removed. Mitochondrial and ribosomal genes were excluded due to their disproportionate expression. The filtered data were normalized to a total of 1 × 10^6^ transcripts per cell and log-transformed to stabilize variance. Highly variable genes were selected (minimum mean = 0.5, maximum mean = 8, minimum dispersion = 0.5). Principal component analysis (PCA) was applied, and the top 40 principal components were used for neighborhood graph construction and clustering with the Leiden algorithm (resolution = 1). Uniform Manifold Approximation and Projection (UMAP) was used for visualization in two dimensions. Cluster marker gene identification and differential expression analysis between WT and E76K or E76R mutants were performed using the Wilcoxon rank-sum test.

### MNase digestion

Global chromatin accessibility was assessed with micrococcal nuclease (MNase) digestion. A total of 1 × 10^6^ freshly harvested cells were resuspended with modified RIPA buffer (50 mM Tris-HCl pH 8.0, 1% NP-40, 0.25% sodium deoxycholate), and the pellet was resuspended with 4 Kunitz U/100 ml MNase (NEB, M0247) and incubated at 37 °C for 10–30 min. DNA was extracted using the Quick-DNA Miniprep Kit (Zymo Research, D3024) and analyzed by gel electrophoresis on a 0.8% agarose gel.

### ATAC-seq

ATAC-seq was performed in IMR-90 SV40-T cells expressing WT or E76K using ATAC-seq Kit (Active Motif, 53150). For TGFb treatment, cells were treated with 2 ng/ml TGFb1 24 h before the experiment. Briefly, 1 × 10^5^ cells were washed with PBS, resuspended in ATAC-seq lysis buffer and incubated with the Tagmentation master mix at 37 °C for 30 min in a thermomixer set at 1,000 rpm. Tagmended DNA was purified, and libraries were prepared with i5 and i7 indexes. Size selection was performed using AMPure XP beads (Beckman Coulter, A63881) with a 0.5–1.3× ratio. Libraries were quantified with the KAPA library quantification kit (Roche) and sequenced on a NovaSeq 6000 (Illumina) at the UC San Diego IGM Genomics Center.

Raw sequencing reads were assessed for quality using FastQC (v0.10.0). Adapter sequences and low-quality bases were trimmed with Trim Galore (v0.3.0), and the resulting clean reads were aligned to the GRCh38 reference genome using Bowtie2 (v2.4.2). Aligned reads were sorted and indexed with SAMtools (v1.6), and PCR duplicates were identified and removed using the MarkDuplicates function in Picard Tools (v2.27.5). Peaks were called using MACS2 (Galaxy v2.2.7.1+galaxy0) from all conditions with or without TGFb treatment, and differential peak detection was performed using featureCounts (Galaxy v2.0.1+galaxy2) followed by DESeq2 (Galaxy v2.11.40.8+galaxy0) across 114,678 peak regions. Gene annotation was performed using GREAT^71^ (v4.0.4), assigning each peak to the nearest genes within ± 5 kb or up to 100 kb. Plot profiles were generated using deepTools computeMatrix (Galaxy v3.5.4+galaxy0) followed by plotProfile (Galaxy v3.5.4+galaxy0).

### SLAM-seq

SLAM-seq^72^ was performed in IMR-90 SV40-T cells expressing WT or mutant H2B using the SLAMseq Kinetics Kit – Anabolic Kinetics Module (Lexogen, 061.24), according to the manufacturer’s protocol. A total of 2 × 10^5^ cells were seeded in a well of a 6-well plate, and media was changed the following day. After an additional day, cells were treated with 100 mM 4-thiouridine (4sU) for 6 h to label newly synthesized RNA. Total RNA was extracted using the Quick-RNA Miniprep Kit (Zymo Research, R1055), followed by alkylation with iodoacetamide (IAA) to induce T>C conversions. RNA libraries were prepared using the QuantSeq 3′ mRNA-Seq V2 FWD Library Generation Module with Lexogen UDI 12 nt Set A1 and amplified using the Library Amplification Module. Libraries were quantified with the KAPA Library Quantification Kit (Roche) and sequenced on a NovaSeq 6000 (Illumina) at the UC San Diego IGM Genomics Center.

Reads were trimmed for low-quality bases (Q<20) using Trimmomatic (Galaxy v0.38.0) and aligned to the GRCh38 reference genome. T>C conversions were quantified using the SLAM-DUNK pipeline (Galaxy v0.4.3+galaxy1), which includes filtering, mapping, mutation calling and quantification modules. Differential expression analysis was performed using DESeq2 (Galaxy v2.11.40.8+galaxy0). Transcripts with significant changes in T>C conversion rates were considered transcriptionally regulated.

### Worm strain construction and maintenance

CRISPR–Cas9 genome editing was used to introduce the E73K and E73R mutations into the endogenous *his-41* locus of the N2 Bristol background. Strains PHX8148, PHX8245 and PHX8202 were generated by Suny Biotech. PHX8148 is a wild-type strain in which the *his-41* E73 codon was replaced with E73E (PHX8148: AATTGCTTCGGAA). The E73K mutation was introduced into PHX8245 (*his-41*(syb8245)); PHX8245: AATTGCTTCGAAA), and the E73R mutation was introduced into PHX8202 (*his-41*(syb8202)); PHX8202: AATTGCTTCGAGA). Worms were maintained on nematode growth medium (NGM) agar plates seeded with *Escherichia coli* strain OP50 at 20 °C. The N2 Bristol strain was obtained from the Caenorhabditis Genetics Center (CGC).

### Lifespan assay in worms

Lifespan assays were conducted following the protocol previously detailed^73^. In brief, L1 age-synchronized worms were plated into 96-well microtiter plates at a volume of 120 ml per well. At the L4 larval stage, animals were sterilized by adding FUDR to each well to a final concentration of 0.12 mM in a total volume of 150 mL. Worm populations were assessed for viability every 2–3 days after shaking plates for 5 min on a microtiter plate shaker to stimulate movement. Plates were stored in incubators maintained at 20 °C. Lifespan analysis was performed using STATA.

### RNA extraction from worms and RNA-seq data analysis

Worm populations were cultured in 15 cm dishes (three dishes per strain and time point), each containing 20 mL of S-complete medium. Worms were maintained at a density of 100 worms/ml and fed OP50 bacteria (ampicillin/carbenicillin-resistant) at a concentration of 4 mg/ml. To prevent contamination, Fungizone (0.1 mg/ml) and carbenicillin (50 mg/ml) were added to the medium. At the L4 stage, FUDR was added to sterilize the animals at a final concentration of 0.12 mM. Worms were harvested at the indicated time points, suspended in minimal PBS and stored at −80 °C overnight or until extraction. For lysis, worms were disrupted in TRIzol (Invitrogen, 15596–026) with zirconium and glass beads using a tissue homogenizer. RNA was extracted by phenol-chloroform separation and further purified using the RNeasy Plus Micro Kit (Qiagen, 74034).

To assess accelerated aging signatures, age-associated genes were first defined by differential expression between WT day 3 and day 10 adults. Genes upregulated with age in WT and already elevated in E73R mutants at day 3 were classified as accelerated age-up genes (n = 645). Conversely, genes downregulated with age in WT and already reduced in E73R mutants at day 3 were defined as accelerated age-down genes (n = 1089). Mean expression levels of these gene sets were calculated per condition, with 95% confidence intervals estimated as mean ± 1.96 × standard error. Gene annotation and functional enrichment analysis of the accelerated age-up and age-down gene sets were performed using Metascape^74^.

### Generation and maintenance of transgenic flies

Transgenes comprising 5×UAS/mini_Hsp70/H2B (His2B:CG17949, NM_165381.4; WT or E73K) Cterminally fused to a Flag tag were generated by VectorBuilder in an attB-containing plasmid backbone and integrated into genomic attP landing sites in *Drosophila melanogaster* via ΦC31 integrase (BestGene Inc.). Ubiquitous expression was achieved by crossing to Act5C-GAL4 drivers (Bloomington Drosophila Stock Center, stock #25374). Flies were maintained on a standard yeast, corn starch, and molasses diet (10% yeast, 12% sugar, 1.5% agar) at 25 °C under a 12 h light/dark cycle and controlled humidity.

### Fly lifespan and locomotor performance assays

Lifespan assays were performed, as previously described^75^. Between 140 and 365 flies per sex and genotype were housed at 25 flies per vial. Flies were transferred to fresh food every 2–3 days, and survival or censoring was recorded at each transfer. Kaplan–Meier survival curves were generated in GraphPad Prism, and significance was assessed by log-rank test.

Locomotor activity was assayed by negative geotaxis at three time points during lifespan, as previously described^76^. Groups of 3–27 flies were tapped to the bottom of the vial, and the fraction ascending above 2 cm within 10 s was scored. Each cohort was tested in triplicate, and four independent cohorts per sex and genotype were analyzed.

### Yeast strain construction and maintenance

Wildtype or mutant (*htb1* D71N, E79K or E79R) copies of the *HTA1*-*HTB1* gene cassette were integrated into the *HTA1*-*HTB1* locus of the *Saccharomyces cerevisiae* histone shuffle strain FY406 (MATa (*hta1*-*htb1*)Δ::LEU2 (*hta2*-*htb2*)Δ::TRP1 *lys2*-128 *leu2*Δ1 *ura3*-52 *trp1*Δ63 *his3*Δ200 pSAB6[URA3, CEN, ARS, *HTA1*-*HTB1*]) using standard transformation protocols. Transformants were selected on 5-fluoroortic acid, genotyped, and the sequence of *htb1* was confirmed by Sanger sequencing. Yeast strains were cultured in YPD medium at 30 °C, and growth was monitored by measuring optical density at 600 nm (OD_600_) at regular intervals to assess proliferation rates.

### Replicative lifespan and stress tolerance assays in yeast

Replicative lifespan was measured using a microfluidics-based platform adapted from the HYAA-Chip protocol^77^. Briefly, filter-sterilized YPD medium was loaded into an AD-Chip at 20 mL/min using 10 mL syringes (BD Biosciences) driven by a KDS-230 pump (KD Scientific) and maintained at 1 mL/min. Yeast cells were grown to mid-log phase, diluted 1:20 and manually loading into the AD-Chip. Multiposition time-lapse imaging was performed using an EVOS FL Auto system equipped with a 30 °C environmental chamber (Thermo Fisher), a 20 × objective and transmitted light optics. Images (three per channel) were acquired every 15 min for 65 h. Image series were analyzed using ImageJ (National Institutes of Health), and cell divisions were manually counted for at least 50 mother cells per strain. Replicative lifespan was defined as the mean number of daughter cells produced. Lifespan differences were assessed using a two-sided Wilcoxon rank-sum test.

Stress tolerance assays were performed using stationary-phase cells, as growth phase can influence stress sensitivity. Cells were plated onto YPD agar or YPD supplemented with stress-inducing agents and incubated under the control condition at 30 °C for 2 days or under stress conditions: heat shock at 42 °C for 16 h or UV irradiation (300 J/m^2^), followed by incubation at 30 °C until 2 days; DNA-damaging agents, including camptothecin (CPT) and methyl methanesulfonate (MMS) for 2 days; and low temperature at 15 °C for 5 days. Growth and survival were assessed by spotting 1:10 serial dilutions starting from 2 OD_600_ and imaging plates after incubation.

### Statistical analysis

No statistical method was used to predetermine sample size. Sample sizes for cell culture experiments were determined empirically for each experiment. Sample sizes for animal experiments were based on pilot studies and were similar to those used previously in comparable worm and fly experiments. Statistical analyses were performed using GraphPad Prism (v9), Microsoft Excel, STATA and R- or Python-based tools, except for sequencing-based analyses. Quantitative data are displayed as mean ± s.d. and represented as error bars unless otherwise indicated. Results from each group were averaged and used to calculate descriptive statistics. *P*-values < 0.05 were considered statistically significant. For cell culture and animal experiments, pairwise comparisons were performed using two-tailed Student’s *t*-tests, assuming equal variance between groups (Fig. 1b,c; 5a,e–j; Extended Data Fig. 1e,i; 2a; 8f, h–j) or one-way ANOVA followed by Dunnett’s post-hoc test to compare each mutant with controls (Fig. 2b,c; Extended Data Fig. 6g). Survival analyses were performed using log-rank (Mantel–Cox) tests, and only adjusted *P*-values are reported (Fig. 4b,k; Extended Data Fig. 7j; 8d). For *C. elegans* mean lifespan comparisons, one-way ANOVA was used to assess group differences, followed by Tukey’s HSD-adjusted emmeans for pairwise comparisons (Fig. 4b). For locomotor performance assay, data were analyzed using two-way ANOVA (genotype × age) separately for each sex, followed by post hoc pairwise comparisons between the Control/WT group and mutant at individual ages using emmeans with Tukey’s HSD adjustment (Fig. 4l). Sequencing data were analyzed using standard pipelines. Differential gene expression was assessed using DESeq2, with genes showing FDR < 0.05 considered differentially expressed for mRNA-seq and differentially accessible for ATAC-seq (Fig. 4d; Extended Data Fig. 3a; 6c). For SLAM-seq, genes with FDR < 0.1 were considered differentially expressed (Extended Data Fig. 6h). GSEA was performed using MSigDB Hallmark and cell type signature databases, including Tabula Muris Senis aging signatures, with Desktop GSEA (v4.4.0). IPA (Qiagen) used a right-tailed Fisher’s exact test to determine statistical significance of upstream regulator enrichment. GSEA FDRs and IPA *Z*-scores are shown without a statistical cut-off to assess data trends unless otherwise indicated. Expression *Z*-scores were calculated using log_2_-transformed expression values followed by sample-wise *Z*-score normalization (mean = 0, SD = 1) (Fig. 3e; Extended Data Fig. 5c). To assess the relationship between chromatin dynamics and gene expression (Fig. 3f), Pearson correlation coefficients and linear regression lines were computed using GraphPad Prism. Estimated immune cell fractions from CIBERSORTx were compared between control and mutant groups using the Wilcoxon rank-sum test (unpaired, two-tailed) for each cell type. For scRNA-seq analysis, clustering and dimensionality reduction were performed using Seurat, and differential expression was assessed using Wilcoxon rank-sum tests with an FDR cutoff of 0.05 for cluster comparisons (Extended Data Fig. 5e). Differential expression of genes across clusters were calculated using log_2_ fold change and FDR from Wilcoxon rank-sum tests (Extended Data Fig. 5g). Investigators were double-blinded when measuring colony numbers in HSPC differentiation assays (Fig. 2b). Otherwise, investigators were not blinded during cell culture experiments, animal experiments or outcome assessment.

## Supplementary Material

Supplementary Files

This is a list of supplementary files associated with this preprint. Click to download.

- ExtendedDataFigtablelegends.docx
- SupplementaryTable1.xlsx
- XXXXXXHiroshi2025.10.20Ex2.pdf
- XXXXXXHiroshi2025.10.20Ex5.pdf
- XXXXXXHiroshi2025.10.20Ex4.pdf
- XXXXXXHiroshi2025.10.20Ex3.pdf
- XXXXXXHiroshi2025.10.20Ex6.pdf
- XXXXXXHiroshi2025.10.20Ex1.pdf
- XXXXXXHiroshi2025.10.20Ex7.pdf
- XXXXXXHiroshi2025.10.20Ex8.pdf

## Data Availability

All sequencing data have been deposited in GEO under accession codes: GSE307377 (mRNA-seq of human donors), GSE307414 (mRNA-seq of HSPCs), GSE307475 (mRNA-seq of IMR-90 SV40-T), GSE307833 (mRNA-seq of *C. elegans*), GSE307869 (ATAC-seq of IMR-90 SV40-T), GSE308172 (SLAM-seq of IMR-90 SV40-T), GSE308173 (scRNA-seq of IMR-90 SV40-T) and GSE308174 (mRNA-seq of mouse tissues). All other raw data can be obtained by contacting the corresponding authors.
